# A GBD 2021 study of Alzheimer’s disease and other dementias attributable to metabolic risk factors and forecasts to 2045 in China

**DOI:** 10.3389/fpubh.2025.1575906

**Published:** 2025-04-01

**Authors:** Meng’en Zhu, Zhimin Bi, Shaoqiong Zhou, Wei Li

**Affiliations:** ^1^Department of Geriatrics, Union Hospital, Tongji Medical College, Huazhong University of Science and Technology, Wuhan, China; ^2^Department of Nephrology, Tongren Hospital of Wuhan University (Wuhan Third Hospital), Wuhan University, Wuhan, China

**Keywords:** Alzheimer’s disease and other dementias, metabolic risks, high fasting plasma glucose, high body mass index, China’s burden of disease

## Abstract

**Background and aims:**

High fasting plasma glucose (FPG) and body mass index (BMI) are recognized as significant metabolic risk factors for Alzheimer’s disease (AD) and other dementias. This study assesses the burden of AD and other dementias attributable to these risks in China using Global Burden of Disease (GBD) 2021 data.

**Methods:**

We estimated deaths, disability-adjusted life-years (DALYs), years lived with disability (YLDs), and age-standardized rates of mortality (ASMR), DALYs (ASDR), and YLDs (ASYR) by age and sex. Temporal trends were analyzed via the average annual percentage change (AAPC), and Bayesian age-period-cohort (BAPC) models were applied to evaluate the effects of age, period, and cohort.

**Results:**

In 2021, China recorded 76,239.36 deaths (95% UI: 2,528.26–259,225.86) from AD and other dementias due to metabolic risks, a 4.7-fold increase from 1990. Females experienced more metabolic risk-related deaths [51,844.08 (95% UI, 1,457.44, 177,037.05)] than males. The ASMR, ASDR, and ASYR showed continuous increases from 1990 to 2021, with AAPC values of 1.03, 1.31, and 1.98%, respectively. A significant increasing trend was observed across age groups from 40 to 95 years, with percentages above 0. Females presented relatively higher risks than males after 1997–2001 and within the birth cohort groups of 1957–1966. The disease burden due to HBMI is expected to rise, while that due to HFPG will decline, notable sex will persist until 2045.

**Conclusion:**

Monitoring trends is crucial for interventions to reduce the future disease burden, particularly among women and older populations in China, to guide healthcare resource allocation effectively.

## Introduction

Dementia is emerging as an urgent public health issue in China because of changes in aging populations along with increasing longevity. It carries profound burdens for individuals and their households, economies of healthcare services and whole communities (1). The number of people with Alzheimer’s disease (AD) and other dementias was more than 9.5 million in 2015 and is expected to grow to 35 million by 2050 in China (2).

In addition to age, approximately 40% of global dementia cases are attributable to 12 modifiable risk factors, including hypertension, obesity, diabetes, and lack of physical activity (3, 4). Although previous studies reported the global spatiotemporal patterns of AD and other dementias burdens caused by all related factors, this overall pattern could not accurately represent the disease burden attributable to metabolic risks in China. Metabolic risk factors, including high fasting plasma glucose (HFPG) and high body-mass index (HBMI), have been hypothesized to play important roles in the development and progression of AD and other dementias (1, 5). While no effective therapies or new drugs are available, growing evidence has suggested that up to one-third of dementia cases could be prevented or delayed by targeting controllable risk factors (6). Therefore, assessing the current status and possible trajectory of the burden of AD and other dementias attributable to HFPG and HBMI is crucial for preventing and treating AD and other dementias in the future.

Research has underscored that the surge in the ASMR of AD and other dementias attributable to HBMI is markedly more significant, particularly affecting the female population, which has heightened in intensity in China (7). Comprehensive research has yet to evaluate the long-term trends of AD and other dementias burdens attributable to HFPG and HBMI in China, with a particular focus on the influences of age and sex. Utilizing GBD 2021 data, this study presents the long-term trends of the disease burden attributable to HFPG and HBMI and predicts their trajectory until 2045. This analysis is crucial for developing targeted health policies and prevention strategies that consider age- and sex-specific risks. This research is designed to optimize health outcomes and medical resource allocation in China, with a focus on demographic groups disproportionately affected by AD and other dementias (8).

## Materials and methods

### Data sources

The specific data analyzed in this study were extracted from the updated online Global Health Data Exchange Query Tool, which includes data on the burden of AD and other dementias attributable to metabolic risks in China (9).

The GBD 2021 study provides an extensive description of the detailed methods used for gathering, processing, and producing data. It is recognized as a high-quality dataset where missing values have been meticulously and skillfully addressed. Numerous advanced studies have leveraged data from the GBD 2021. The primary indicators for measuring the burden of disease in these studies include the age-standardized mortality rate (ASMR, per 100,000), the age-standardized disability-adjusted life year rate (ASDR, per 100,000), and the disability-adjusted life years (DALYs). The ASMR is defined as the weighted average of mortality rates at a specific age per 100,000 individuals, with the weights determined by the proportion of the population in the corresponding age group according to the World Health Organization (WHO) standard population. The ASDR is calculated by applying age-specific weights to the DALYs, reflecting the different age distributions within the population. DALYs represent the sum of years of life lost due to premature mortality (YLL) and the years lived with disability (YLDs) as a result of diseases or health conditions. YLL refers to the years of life lost due to early death, whereas YLD accounts for any short-term or long-term loss of healthy life years due to disability. For further clarification and details on these definitions, the WHO website provides a comprehensive resource (accessed on 18 November 2024)^1^ (10, 11).

Since there were few dementia cases in people under 40 years of age, only cases in people over 40 years of age were analyzed. The age-standardized rates (ASRs) stratified by age (5-year age groups of patients aged 40–94 years and ≥ 95) and sex (both male and female) were evaluated for the epidemiological burden and trends of AD and other dementias due to metabolic risks in China from 1990 to 2021 by extracting annual deaths, DALYs, YLDs, and their corresponding 95% uncertainty intervals (UIs) (1, 10, 12).

### Definitions of AD and other dementias

Dementia is defined as a chronic syndrome characterized by progressive and degenerative cognitive dysfunctions that interfere with activities of daily living and is usually accompanied by behavioral and psychological symptoms (13). The type of dementia mentioned in this article referred to AD and other dementias (13), which were identified according to the International Classification of Diseases version 10 codes: F00–F03, G30-G31 (14).

### Definitions of HFPG and HBMI

We ultimately included HFPG and HBMI, for which sufficient evidence exists to merit inclusion as metabolic risk factors for dementia in the GBD 2021. In the GBD, HFPG was measured as the mean fasting plasma glucose in the population, where fasting plasma glucose was a continuous exposure in mmol/L. HFPG was defined as any level of fasting plasma glucose above the theoretical minimum-risk exposure level (4.8–5.4 mmol/L). HBMI for adults (aged 20+) was defined as a high body mass index (BMI) greater than 20–25 kg/m^2^ and thresholds from the International Obesity Task Force standards were used (15).

### Statistical analysis

To evaluate the burden and trends of AD and other dementias attributable to HFPG and HBMI from 1990 to 2021, we used joinpoint regression analysis to calculate the annual percentage change (APC), the average annual percentage change (AAPC), and the corresponding 95% confidence interval (CI). Joinpoint version 4.9.0.1 software developed by the National Cancer Institute of the United States was used for joinpoint regression analysis. As described by Kim et al. (16). There is an increasing trend of ASR if the estimated APC/AAPC and the lower limit of the 95% CI are both greater than 0. Conversely, a decreasing trend is exhibited if the estimated APC/AAPC and the upper limit of the 95% CI are both less than 0. Otherwise, the ASR is considered stable.

The age-period-cohort model was applied to estimate the effects of age, period, and cohort on the temporal trends in mortality, DALYs, and YLDs rates of AD and other dementias attributable to HFPG and HBMI (17). Mortality cases, DALYs cases and YLDs cases and population data for AD and other dementias attributable to HFPG and HBMI were arranged into 12 continuous 5-year age groups from 40–44 years to 90–94 years and the 95 plus years, six consecutive 5-year periods from 1992–1996 to 2017–2021 and 17 successive 5-year birth cohorts ranging from 1892–1901 to 1972–1981. We estimated the rate ratios (RRs) of mortality, DALYs and YLDs rates attributed to AD and other dementias in different periods and cohorts relative to the reference points specified age and sex groups and calendar time periods. We also computed the net drift, local drifts, and longitudinal age curve of mortality, DALYs rate and YLDs rate for AD and other dementias due to metabolic risks. Net drift represents the annual percentage change in age-adjusted mortality, DALYs rate and YLDs rate for AD and other dementias due to metabolic risks over time. Local drifts represent the annual percentage changes in the mortality, DALYs rate and YLDs rate for AD and other dementias due to metabolic risks in each age group over time. The longitudinal age curve represents the expected age-specific rates in the reference cohort adjusted for period effects. The estimated parameters were determined via the age-period-cohort Web Tool from the National Cancer Institute. Wald chi-square tests were adopted for the significance of the estimable parameters and functions (18).

The burden of AD and other dementias due to metabolic risks (HFPG and HBMI) from 2022 to 2045 in China was predicted with a Bayesian age–period–cohort (BAPC) model of integrated nested Laplace approximations (R packages, BAPC, Stata 16 MP-64 and INLA), assuming the inverse gamma prior distribution of age, period, and cohort effects and applying a second-order random walk (RW2) to adjust for excessive dispersion (18). To present our forecast results with greater clarity, we set up a baseline reference, negative reference, and positive reference. The baseline reference reflects the 2021 statistics for deaths, DALYs, and YLDs from AD and other dementias due to metabolic risks. The negative reference projects a 1% annual increase in these metrics from 2021, whereas the positive reference envisions a 1% annual decrease. Our posterior distributions are derived from 2021 Chinese standard population data, with projected rates based on the anticipated future population of China (19).

Other analyses and visualizations were performed via R software (version 4.2.2). A *p* value <0.05 was considered to indicate statistical significance.

## Results

### Descriptive analysis

All-age cases, ASR (per 100,000) and AAPC of deaths, DALYs and YLDs for AD and other dementias attributable to metabolic risks, HFPG and HBMI in China, 1990–2021 are presented in Table 1, which clearly revealed that there was a higher disease burden in 2021 than in 1990. For instance, the number of deaths increased from 13,384.89 to 76,239.36 in 2021, indicating a 4.7-fold increase from 1990; the number of DALYs cases increased from 295,648.37 in 1990 to 1,593,832.84 in 2021, with a 4.39-fold increase; and the number of YLDs cases increased from 86,029.02 in 1990 to 544,875.44 in 2021, with a percentage change of 533%. More metabolic risk-related deaths were reported among females [51,844.08 (95% UI: 1,457.44, 177,037.05)] than among males [24,395.27 (95% UI: 1,154.16, 86,595.49)] in 2021. Compared with males, females had higher ASMRs of AD and other dementias attributable to metabolic risks [5.27 (95% UI: 0.15, 18.11) and 3.75 (95% UI: 0.18, 12.76), respectively]. Similar trends were found in the disease burden associated with HFPG and HBMI (Table 1).

**Table 1:** The disease burden of AD and other dementias attributable to metabolic risks cases and rates in 1990 and 2021, and the trends from 1990 to 2021, in China.

		Cases of people with AD and other dementias due to metabolic risks			Rates of people with AD and other dementias due to metabolic risks		
Disease burden due to risk factors	Sex	1990 (95% UI)	2021 (95% UI)	Percentage change (100%)	1990_ASR per 100,000	2021_ASR per 100,000	AAPC (95% CI)
Deaths							
Metabolic risks	Male	4,368.23 (415.07–15,433.82)	24,395.27 (1,154.16–86,595.49)	4.58	2.75 (0.27–9.34)	3.75 (0.18–12.76)	1.03 (0.99–1.06)
Metabolic risks	Female	9,016.65 (902.64–30,964.56)	51,844.08 (1,457.44–177,037.05)	4.75	3.79 (0.38–12.83)	5.27 (0.15–18.11)	1.09 (1.05–1.13)
Metabolic risks	Both	13,384.89 (1,334.29–46,875.3)	76,239.36 (2,528.26–259,225.86)	4.7	3.43 (0.35–11.59)	4.69 (0.17–15.91)	1.03 (0.99–1.07)
HBMI	Male	59.47 (−267.15 to 1,368.8)	5,395.16 (−73.65 to 25,170.76)	89.72	0.01 (−0.18 to 0.88)	0.81 (−0.01 to 3.89)	14.11 (14.03–14.21)
HBMI	Female	333.8 (−414.11 to 3,731.97)	15,461.16 (−1,159.37 to 70,614.84)	45.32	0.1 (−0.21 to 1.47)	1.55 (−0.11 to 7.19)	9.25 (9.2–9.31)
HBMI	Both	393.27 (−662.39 to 5,193.32)	20,856.32 (−1,247.33 to 95,455.09)	52.03	0.06 (−0.2 to 1.22)	1.26 (−0.07 to 5.88)	9.98 (9.93–10.03)
HFPG	Male	4,318.74 (164.96–14,364.66)	19,916.45 (800.12–65,530.13)	3.61	2.74 (0.1–8.84)	3.07 (0.12–9.8)	0.42 (0.38–0.45)
HFPG	Female	8,732.88 (354.22–28,251.96)	38,921.79 (1,534.73–123,451.56)	3.46	3.71 (0.15–11.69)	3.98 (0.16–12.72)	0.24 (0.19–0.29)
HFPG	Both	13,051.62 (519.18–43,548.01)	38,921.79 (1,534.73–12,3451.57)	1.98	3.38 (0.13–10.63)	3.64 (0.14–11.53)	0.29 (0.24–0.33)
DALYs							
Metabolic risks	Male	103,445.12 (16,498.33–318,355.07)	536,880.29 (46,607.99–1,526,223.58)	4.19	46.47 (7.47–138.41)	68.15 (6.18–191.84)	1.25 (1.22–1.28)
Metabolic risks	Female	192,203.24 (29,919.24–567,420.44)	1,056,952.55 (52,188.12–2,955,600.55)	4.5	65.44 (10.09–192.44)	101.43 (5.28–284.96)	1.41 (1.38–1.44)
Metabolic risks	Both	295,648.37 (48,704.02–890,869.04)	1,593,832.84 (99,393.63–4,550,014.52)	4.39	58.2 (9.31–172.43)	87.41 (5.75–251.89)	1.31 (1.29–1.34)
HBMI	Male	1,530.99 (−6,443.3 to 35,325.56)	125,811.81 (−2,825.76 to 489,745)	81.18	0.21 (−3.08 to 14.11)	15.28 (−0.27 to 61.44)	14.75 (14.63–14.88)
HBMI	Female	8,818.63 (−8,359.18 to 74,546.37)	340,916.87 (−37,073.64 to 1,293,098.82)	37.66	2.14 (−3.26 to 24.38)	32.02 (−3.22 to 123.76)	9.05 (8.99–9.13)
HBMI	Both	10,349.62 (−14,418.8 to 112,004.91)	466,728.68 (−36,467.55 to 1,800,202.16)	44.1	1.29 (−3.17 to 20.53)	24.75 (−1.75 to 98.65)	9.94 (9.88–10)
HFPG	Male	102,135.91 (5,583.57–292,581.67)	432,109.75 (25,175.19–1,174,803.3)	3.23	46.3 (2.55–129.67)	55.42 (3.12–148.24)	0.61 (0.58–0.65)
HFPG	Female	184,629.39 (10,158.26–510,426.45)	771,929.66 (46,281.17–2,061,089.58)	3.18	63.61 (3.5–175.09)	74.65 (4.46–198.27)	0.53 (0.5–0.57)
HFPG	Both	286,765.3 (15,741.84–806,461.65)	1,204,039.42 (70,781.95–3,184,253.24)	3.2	57.1 (3.15–157.09)	66.72 (3.91–177.01)	0.53 (0.49–0.56)
YLDs							
Metabolic risks	Male	29,493.70 (6,458.45–66,087.56)	176,129.39 (20,454.42–404,087.78)	4.97	11.94 (2.67–26.59)	20.98 (2.55–48.77)	1.80 (1.76–1.84)
Metabolic risks	Female	56,535.32 (11,935.89–129,826.48)	368,746.05 (26,668.26–874,014.53)	5.52	17.91 (3.93–40.70)	34.68 (2.62–82.19)	2.12 (2.05–2.16)
Metabolic risks	Both	86,029.02 (18,394.35–195,913.13)	544,875.44 (48,522.53–1,287,160.64)	5.33	15.54 (3.45–34.78)	28.76 (2.68–67.83)	1.98 (1.93–2.02)
HBMI	Male	7.70 (−3,313.38 to 7,945.94)	40,355.71 (−1,516.58 to 123,317.36)	5,240	−0.13 (−1.54 to 2.91)	4.57 (−0.12 to –14.39)	26.99 (24.74–28.62)
HBMI	Female	2,034.38 (−4,192.89 to 18,112.17)	119,046.13 (−14,671.51 to 383,598.04)	57.52	0.40 (−1.50 to 5.12)	10.95 (−1.27 to 35.44)	11.16 (11.05–11.25)
HBMI	Both	2,042.09 (−7,232.21 to 25,265.27)	159,401.84 (−15,295.82 to 511,229.01)	77.06	0.16 (−1.51 to 4.32)	8.11 (−0.72 to 26.00)	13.37 (13.25–13.48)
HFPG	Male	29,477.97 (2,512.78–64,367.84)	142,527.23 (12,860.36–311,071.93)	3.84	12.04 (2.09–26.40)	17.18 (1.55–37.63)	1.14 (1.10–1.17)
HFPG	Female	54,766.23 (4,704.27–119,256.56)	269,353.58 (24,003.37–585,586.77)	3.92	17.56 (1.53–38.55)	25.53 (2.27–55.98)	1.15 (1.10–1.20)
HFPG	Both	84,244.20 (7,217.04–183,717.25)	411,880.82 (36,863.70–893,080.46)	3.89	15.40 (1.35–34.02)	21.98 (1.97–48.14)	1.12 (1.07–1.15)

AD, Alzheimer’s disease; DALYs, disability-adjusted life-years; YLDs, years lived with disability; ASR, age-standardized rate; HBMI, High body-mass index; HFPG, High fasting plasma glucose; UI, uncertainty interval; AAPC, average annual percentage change; CI, confidence interval.

### Comparison by age and sex in 1990 and 2021

**Figure 1:**
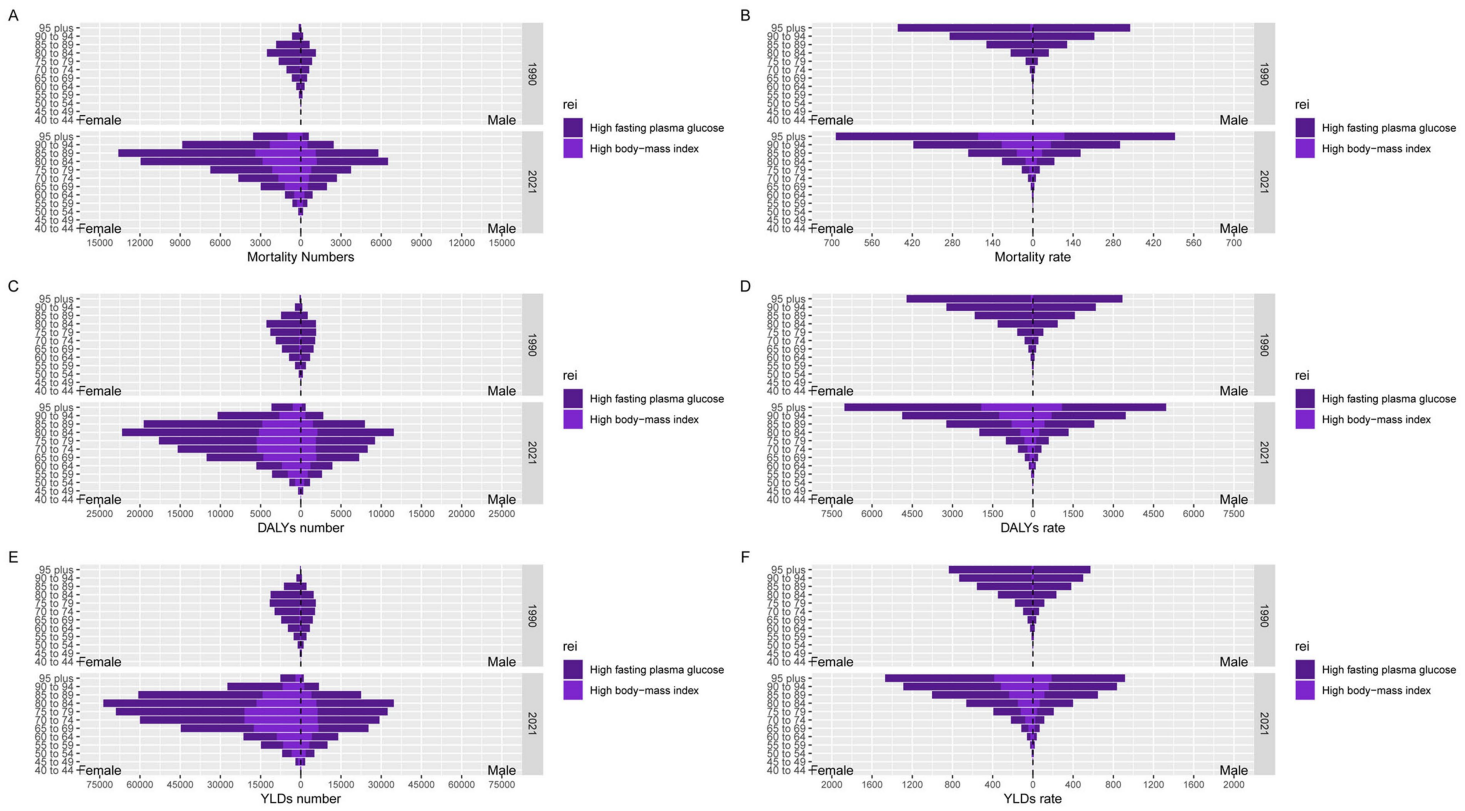
Numbers and rates of mortality, DALYs and YLDs for AD and other dementias due to HFPG and HBMI by sex and age group in 1990 and 2021 in China. **(A)** Mortality number; **(B)** Mortality rate; **(C)** DALYs number; **(D)** DALYs rate; **(E)** YLDs number; **(F)** YLDs rate.

Since 1990, the burden of AD and other dementias attributable to HFPG and HBMI has increased with age. The disease burden attributable to HFPG and HBMI was even greater in females than in males both in 1990 and 2021 in China. The age group with the highest HFPG- and HBMI-related AD and other dementias deaths was people aged 85–89 years in 2021 but 80–84 years in 1990. In both 1990 and 2021, most of the overall AD and other dementias death cases across all age groups were attributable to HFPG. (Figure 1).

### Joinpoint regression analysis

**Figure 2:**
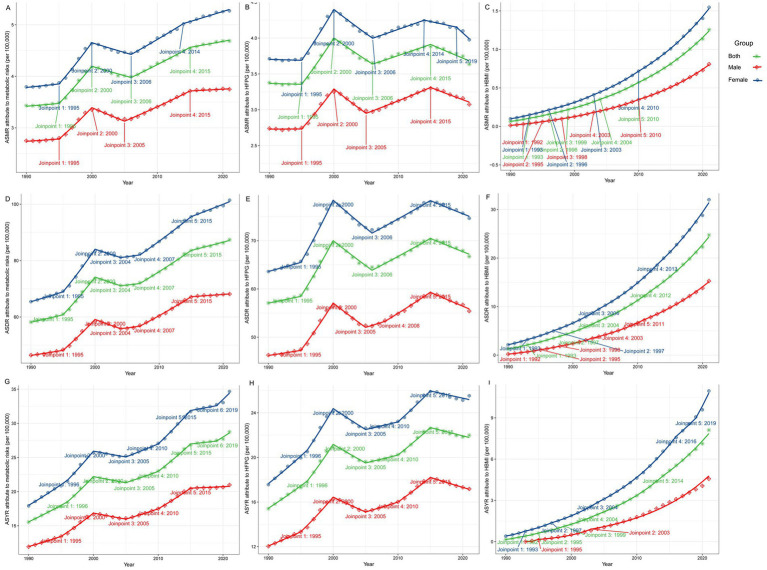
Temporal changes in the ASMR, ASDR and ASYR for AD and other dementias due to metabolic risks by sex in China from 1990 to 2021. **(A)** ASMR attributed to metabolic risks. **(B)** ASMR attributed to HFPG. **(C)** ASMR attributed to HBMI. **(D)** ASDR attributed to metabolic risks. **(E)** ASDR attributed to HFPG. **(F)** ASDR attributed to HBMI. **(G)** ASYR contributes to metabolic risks. **(H)** ASYR attributed to HFPG. **(I)** ASYR attributed to HBMI (*p < 0.05).

As shown in Table 1, Supplementary material 1, and Figure 2, the ASMR, ASDR, and ASYR for AD and other dementias due to metabolic risks increased from 1990 to 2021 in China among both sexes, with AAPC values of 1.03% (95% CI: 0.99, 1.07), 1.31% (95% CI: 1.29, 1.34) and 2.12% (95% CI: 2.05–2.16), respectively. Additionally, the ASMR, ASDR and ASYR for AD and other dementias due to metabolic risks also increased from 1990 to 2021 in China among females, with AAPC values of 1.09% (95% CI: 1.05–1.13), 1.41% (95% CI: 1.38–1.44) and 1.98% (95% CI: 1.93–2.02), respectively, which were higher than those reported in males. Otherwise, the ASMR, ASDR, and ASYR in both sexes due to HFPG showed downward trends after 2015 [APC_2015-2021 for ASMR_ = −1.01% (95% CI: −1.34, −0.71), APC_2015–2021 for ASDR_ = −0.79% (95% CI: −1.05, −0.55), APC_2015–2021 for ASYR_ = −0.66% (95% CI: −0.92, −0.42)]. Additionally, the joinpoint analysis of the ASMR, ASDR and ASYR attributable to HBMI exhibited continuously increasing trends from 1990 to 2021, with AAPC values of 9.25% (95% CI: 9.2, 9.31), 9.05% (95% CI: 8.99, 9.13) and 11.16% (95% CI: 11.05, 11.25), respectively.

### Age-period-cohort model analysis

**Figure 3:**
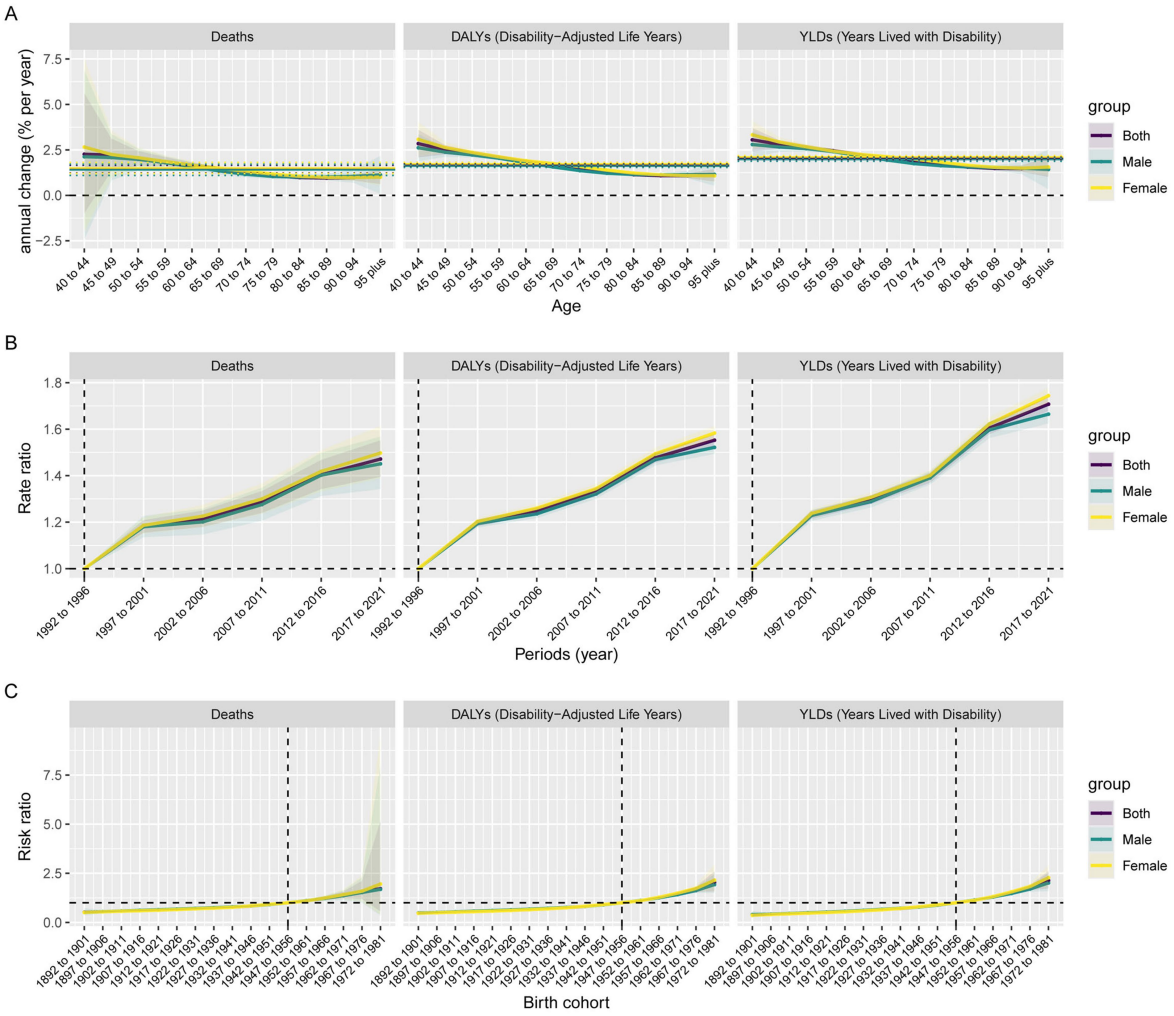
Parameter estimates of age, period, and cohort effects on AD and other dementias burden attributable to metabolic risks by sex in China from 1990 to 2021. **(A)** Local drifts with net drift values; the horizontal solid and dashed lines represent the net drift and its 95% CI, respectively, and the solid curves and shaded areas represent the local drift and their 95% CIs, respectively. **(B)** Rates of mortality, DALYs rate and YLDs rate on AD and other dementias attributable to metabolic risk factors in different periods (period effects) relative to the reference period (1992–1996) (the junction of the solid curves and the dashed lines), adjusted for age and birth cohort effects, and the corresponding 95% CI (shaded area). **(C)** Rates of mortality, DALYs and YLDs on AD and other dementias attributable to metabolic risk factors in different cohorts (cohort effects) relative to the reference birth cohort (birth cohort 1947–1956) (the junction of the solid curves and the dashed lines), adjusted for age and period effects, and the corresponding 95% CI (shaded area). (Different colors represent different sexes. Some were overlapped and too narrow to show).

Across the study period, the annual percentage changes (net drifts) in deaths, DALYs and YLDs for AD and other dementias attributable to metabolic risk factors among males were 1.40 (95% CI: 1.10, 1.71), 1.61 (95% CI: 1.53, 1.68), and 1.97 (95% CI: 1.87, 2.06), respectively. Notably, these rates were lower than those recorded for females (Figure 3A; Table 2). This suggested a significantly increased trend of AD and other dementias burden attributable to metabolic risk factors after excluding the effects of age and birth cohort. Moreover, this trend varied significantly according to age (local drifts), and the percentage was above 0 among all subjects, indicating an increasing trend across all age groups from 40 to 95 years. The age-specific trends for AD and other dementias burden attributable to metabolic risks increased with age among both sexes, males and females, with a rapid increase for those over 65–69 years (Figure 3A; Supplement material 2:Table 1).

**Table 2:** The annual percentage change (net drift) for AD and other dementias deaths attributable to metabolic risk factors, 1990–2021.

	Net drift		
Sex	Deaths due to metabolic risks	DALYs due to metabolic risks	YLDs due to metabolic risks
Both	1.44 (1.23–1.66)	1.66 (1.60–1.73)	2.04 (1.98–2.11)
Male	1.40 (1.10–1.71)	1.61 (1.53–1.68)	1.97 (1.87–2.06)
Female	1.50 (1.20–1.81)	1.73 (1.66–1.81)	2.11 (2.04–2.19)

DALYs, Disability-Adjusted Life Years; YLDs, Years Lived with Disability.

The period effects of AD and other dementias burden attributable to metabolic risks tended to increase for both sexes during the whole study period, suggesting that the burden of AD and other dementias increased over time, with the risk increasing and remaining above 1. Particularly, the period effects showed gradual upward trends for individuals from 2002–2006 to 2012–2016 compared with the reference period (1992–1996). For example, the RR for metabolic risks-related mortality in males increased from 1 to 1.5 during 1990–2021. Additionally, females had relatively higher risks of AD and other dementias attributable to metabolic risks than males did after 1997–2001. Similar period effects on the DALYs and YLDs rates of AD and other dementias due to metabolic risks were observed among individuals (Figure 3B; Supplement material 2:Table 2).

The cohort RR for AD and other dementias burden attributable to metabolic risks showed continuously increasing patterns for both sexes in the observed birth cohorts, especially after the reference cohort (1947–1956), with an RR > 1. Similar trends were observed among males and females in the cohort effects on the DALYs and YLDs rates of AD and other dementias due to metabolic risks. Otherwise, after the birth cohort groups of 1957–1966, females had higher relative risks of disease burden than males. (Figure 3C; Supplement material 2:Table 3).

### Prediction by the BAPC model

**Figure 4:**
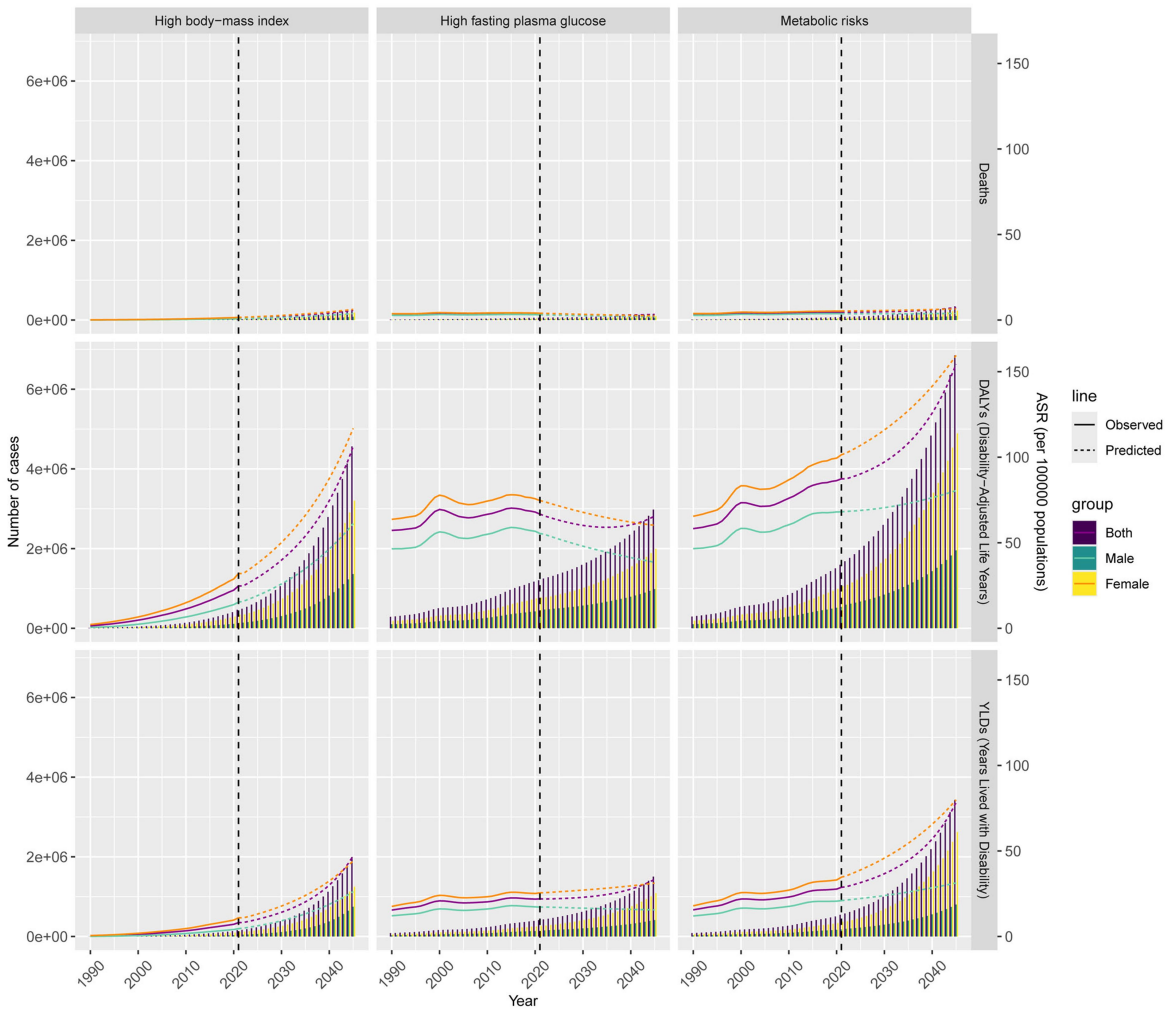
Numbers and ASRs of mortality, DALYs and YLDs for AD and other dementias burden attributable to metabolic risks from 2022 to 2045 by sex.

When we utilized the BAPC model to project the disease burden of AD and other dementias attributable to metabolic risks from 2022 to 2045, the mortality, DALYs and YLDs predictions for HFPG and HBMI showed heterogeneity. These predictions indicate that while the disease burden due to metabolic risks will increase, the disease burden due to HFPG will decline, and notable sex will persist. For instance, the number of all-age mortality cases due to metabolic risks will increase to 343821.34 (95% CI: 0.00, 997836.09) by 2045, and the ASMR will increase to 7.27 (95% CI: 0.00, 21.12). Compared with 2021, the number of all-age cases associated with mortality will increase by 3.51 times, and the ASMR will increase by 55.34%. Additionally, females tend to experience a greater disease burden due to metabolic risks than males. The ASMR is projected to increase to 6.52% for females, whereas for males, it is forecasted to be 3.86%. For both sexes, even as the number of all-age cases increases, the ASMR and ASDR due to HFPG decrease to 3.28 and 65.60%, respectively. Specifically, the disease burden due to HBMI is anticipated to persist in its upward trajectory (Figure 4; Supplement material 3).

## Discussion

This study offers novel insights into the trends of AD and other dementias burdens attributable to metabolic risks in China on the basis of data from the GBD 2021 study. There was an increasing trend in the burden of AD and other dementias due to metabolic risks from 1990 to 2021. In 2021, women had a greater disease burden than men.

In 2021, utilizing an expanded GBD framework, AD and other dementias were among the top 10 contributors to neurological DALYs. HFPG is a significant risk factor for AD and other dementias, accounting for 14.6% of cases (20). The aging population and heightened risk exposure have jointly led to the most significant increase in the attributable burden of risk for obesity and metabolic syndrome-related factors, especially HFPG and HBMI (21).

Since China has a large population of older people, the burden of AD and other dementias increases with age (22). Research indicates that age is a pivotal factor in the risk of AD and other dementias mortality, yet the impact of sex on lifestyle choices and health profiles also significantly shapes these mortality rates. This divergence is deeply rooted in the intrinsic physiological differences between males and females, potentially attributable to sex differences in brain structure, development, function, and biochemistry (7, 22). Additionally, women, with their increased inflammatory response, are more likely to develop diabetes and metabolic syndrome. Globally, women’s average life expectancy is 4.5 years longer than that of men. This extended lifespan, coupled with their heightened vulnerability to certain health conditions, results in women experiencing a greater prevalence of AD-related deaths and imposing a greater disease burden (7). More attention should be given to prevention and postillness care for women and older populations with AD and other dementias (23). Thus, it is essential to take gender-specific factors into account when assessing dementia risk and devising preventive strategies.

In the current study, from 2015 to 2021, ASMR, ASDR, and ASYR due to HFPG significantly decreased, which may be driven by “social determinants” (11). Since 2003, China has made significant efforts in chronic disease management (11). However, the ASMR, ASDR, and ASYR attributed to HBMI increased from 1990 to 2021. First, with rapid economic growth, the consumption levels and lifestyles of Chinese residents have improved rapidly, the exercise time of Chinese residents has rapidly decreased, and sedentary time has gradually increased (11, 22, 24). Second, the 7th National Census of China in 2020 revealed a significant demographic shift, with the population aged 65 years and above constituting 13.5% of the total population, indicating an aging society (25). This rapid aging will drive a continuous increase in the disease burden (11, 22). Additionally, the proliferation of the food industry has diversified food options, with a surge in ultra-processed foods. This trend has steered the traditional Chinese diet toward Western dietary patterns marked by decreased whole-grain consumption and increased intake of red meat, processed meats, sugary beverages, and high-fat foods. Concurrently, the rise in wealth among rural populations has prompted a dietary shift toward more animal products and fats, thereby contributing to an increased obesity rate. These findings indicate that HBMI is an important metabolic risk factor for AD and other dementias burdens in China. Additionally, we found that females had higher AAPCs than males. This may reveal a potential increasing burden of dementia attributable to metabolic risks in females.

Additionally, the impacts of metabolic risk factors (including HFPG and HBMI) on the mortality rate, DALYs rate, and YLDs rate of AD and other dementias usually increase with age. The period effect on the burden of AD and other dementias attributable to metabolic risks showed an upward trend from 1990 to 2021. The RR of the cohort effect on the burden of AD and other dementias due to metabolic risks had increased from earlier to later birth cohorts, suggesting that later birth cohorts had greater disease burdens due to metabolic risks than earlier birth cohorts. Since the Health Sector Reform in 2009, the Chinese government has increased financial investment in public health services and provided measures, including health education and health management services for chronic disease patients (26). Adequate investment in policy development and financial resources is essential for ensuring a high detection rate of fasting glucose levels among individuals in China. This strategic allocation of resources plays a critical role in the early identification and management of diabetes, thereby contributing to the overall health and well-being of the population (27). With the improvement of living standards in China, the dietary structure and entertainment forms of Chinese residents have changed dramatically (26). These changes might increase the risk of suffering from various chronic diseases, such as diabetes and obesity, which are strongly correlated with the onset of AD and other dementias (24, 26, 28, 29). In a previous study, downward trends due to AD and other dementias were found in later birth cohorts because they received better education and had greater awareness of health and disease prevention than earlier birth cohorts. However, vascular risk factors, including diabetes and obesity, are more prevalent in later birth cohorts than in earlier birth cohorts (17). According to the GBD 2021 study, as risk exposure escalates, along with the interplay of metabolic risk factors such as HFPG, high systolic blood pressure, reduced physical activity, and increased sugar-sweetened beverage intake, there is an urgent imperative for interventions aimed at addressing obesity and metabolic syndrome (21). Therefore, improved strategies are needed for later birth cohorts, who face greater risk.

Considering the overall impact on disease burden, the trajectory of attributable burdens, and the robustness of evidence correlating risk factor exposures with specific health outcomes, HBMI and HFPG rank among the top 10 risk factors. For individuals over 50 years of age, metabolic risks continue to predominate (21). Our BAPC showed that the HFPG-related burden will continue to steadily increase until 2045. The HBMI-related burden rapidly increased from 2022 to 2045. China has the largest population of people with dementia in the world (22, 30). Therefore, a multifaceted approach involving heightened public awareness, the promotion of healthier living and dietary choices, and the strategic enhancement of healthcare resource allocation and utilization to significantly reduce the disease burden is essential. And more attention should be given to weight management. The study limitations were mainly as follows: 1. Our study only considered metabolic factors associated with AD and other dementias, without other relevant risk factors. 2. Our research data were not divided into dementias by pathological subtype. 3. The diagnostic criteria, biomarkers, medical records and insurance codes for global dementia have been updated over time, with possible heterogeneity over the past decades. 4. The discrepancy in AD and other dementias burdens between our findings and the real world was inevitable. Therefore, our results from the present study on the epidemiology of AD and other dementias should be interpreted with caution.

## Conclusion

This study revealed that the burden of AD and other dementias attributable to metabolic risks increased from 1990 to 2021 and will increase continuously until 2045 in China. The burden of this disease affected the older adult, especially the females. Additionally, the increasing trends of the disease burden attributable to HBMI were observed from 1990 to 2045 in China. Those findings of this study underscore the urgent need for more effective prevention and management strategies. While there are initiatives in place, enhanced efforts are necessary to address this growing health concern. More efforts are needed to focus on weight management. Additionally, early interventions should be proposed to reduce the disease burden on women and older populations at high risk. Future studies should prioritize exploring the associations between AD and other dementias with metabolic risks, while also assessing the efficacy of targeted inventions on these diseases.

## Data Availability

The original contributions presented in the study are publicly available. This data can be found at: http://ghdx.healthdata.org/gbd-results-tool.

## Footnotes

^1^ www.who.int/data/gho/indicator-metadata-registry
