# Supplementary material for: A GBD 2021 study of Alzheimer’s disease and other dementias attributable to metabolic risk factors and forecasts to 2045 in China
Source: Front Public Health. 2025 Apr 1;13:1575906. doi: 10.3389/fpubh.2025.1575906 (PMC11998917; doi:10.3389/fpubh.2025.1575906)
Supplement: Supplementary file 1 [file Supplementary_file_1.docx]

**Supplementary 1**, Trends in ASR of deaths, DALYs and YLDs of Alzheimer’s disease and other dementias due to metabolic risks in China, 1990-2021

| **Metabolic risks** | **Both** | |  |  | **Male** |  |  |  | **Female** |  |  |  |
| --- | --- | --- | --- | --- | --- | --- | --- | --- | --- | --- | --- | --- |
|  | **Segment** | **Period** | **APC (95%CI)** | **p value** | **Segment** | **Period** | **APC (95%CI)** | **p value** | **Segment** | **Period** | **APC (95%CI)** | **p value** |
| **Deaths** | 1 | 1990-1995 | 0.32(0.08 to 0. 57) | 0.01 | 1 | 1990-1995 | 0.32 (0.1 to 0.54) | 0.005 | 1 | 1990-1995 | 0.36 (0.05 to 0.67) | 0.036 |
|  | 2 | 1995-2000 | 3.85 (3.64 to 4.07) | 0.00 | 2 | 1995-2000 | 4.04 (3.85 to 4.24) | 0.00 | 2 | 1995-2000 | 3.85 (3.61 to 4.15) | 0.00 |
|  | 3 | 2000-2006 | -0.89 (-1.08 to -0.72) | 0.00 | 3 | 2000-2005 | -1.6 (-1.77 to -1.43) | 0.00 | 3 | 2000-2006 | -0.84 (-1.15 to -0.64) | 0.00 |
|  | 4 | 2006-2015 | 1.54 (1.42 to 1.7) | 0.00 | 4 | 2005-2015 | 1.73 (1.64 to 1.84) | 0.00 | 4 | 2006-2014 | 1.6 (1.45 to 1.9) | 0.00 |
|  | 5 | 2015-2021 | 0.5 (0.18 to 0.72) | 0.009 | 5 | 2015-2021 | 0.19 (-0.11 to 0.42) | 0.112 | 5 | 2014-2021 | 0.76 (0.48 to 0.95) | 0.003 |
|  | 6 | … | … | … | 6 | … |  |  | 6 | … |  |  |
| **DALYs** | 1 | 1990-1995 | 0.89 (0.7 to 1.07) | 0.00 | 1 | 1990-1995 | 0.77 (0.58 to 0.98) | <0.001 | 1 | 1990-1995 | 1.04 (0.84 to 1.23) | 0.00 |
|  | 2 | 1995-2000 | 4.06 (3.89 to 4.24) | 0.00 | 2 | 1995-2000 | 4.19 (4 to 4.38) | 0.00 | 2 | 1995-2000 | 4.05 (3.86 to 4.24) | 0.00 |
|  | 3 | 2000-2004 | -1.06 (-1.43 to -0.8) | 0.008 | 3 | 2000-2004 | -1.43 (-1.85 to -1.18) | 0.003 | 3 | 2000-2004 | -0.9 (-1.27 to -0.62) | 0.016 |
|  | 4 | 2004-2007 | 0.37 (-0.46 to 1.39) | 0.13 | 4 | 2004-2007 | 0.54 (-0.5 to 1.58) | 0.123 | 4 | 2004-2007 | 0.39 (-0.4 to 1.62) | 0.12 |
|  | 5 | 2007-2015 | 1.91 (1.78 to 2.12) | 0.00 | 5 | 2007-2015 | 2.13 (2 to 2.34) | 0.002 | 5 | 2007-2015 | 1.93 (1.79 to 2.19) | 0.00 |
|  | 6 | 2015-2021 | 0.7 (0.47 to 0.89) | <0.001 | 6 | 2015-2021 | 0.24 (0.05 to 0.45) | 0.027 | 6 | 2015-2021 | 0.91 (0.66 to 1.11) | 0.00 |
| **YLDs** | 1 | 1990-1996 | 2.92 (2.57 to 3.17) | 0.00 | 1 | 1990-1995 | 2.37 (1.9 to 2.71) | 0.00 | 1 | 1990-1996 | 3.15 (2.6 to 3.43) | 0.00 |
|  | 2 | 1996-2000 | 4.65 (4.22 to 5.23) | 0.00 | 2 | 1995-2000 | 4.64 (4.36 to 5.08) | 0.00 | 2 | 1996-2000 | 4.57 (4.11 to 5.2) | <0.001 |
|  | 3 | 2000-2005 | -0.82 (-1.18 to -0.51) | 0.024 | 3 | 2000-2005 | -1.07 (-1.44 to -0.81) | <0.001 | 3 | 2000-2005 | -0.67 (-1.18 to -0.33) | 0.016 |
|  | 4 | 2005-2010 | 1.5 (0.9 to 1.9) | 0.026 | 4 | 2005-2010 | 1.82 (1.22 to 2.21) | 0.002 | 4 | 2005-2010 | 1.48 (0.75 to 2.09) | 0.016 |
|  | 5 | 2010-2015 | 3.28 (2.85 to 3.78) | 0.00 | 5 | 2010-2015 | 3.24 (2.88 to 3.84) | <0.001 | 5 | 2010-2015 | 3.39 (2.94 to 4.04) | 0.00 |
|  | 6 | 2015-2019 | 0.38 (-0.19 to 0.9) | 0.142 | 6 | 2015-2021 | 0.22 (-0.04 to 0.47) | 0.079 | 6 | 2015-2019 | 0.55 (-0.12 to 1.08) | 0.98 |
|  | 7 | 2019-2021 | 2.17 (1.17 to 2.87) | 0.00 |  |  |  |  | 7 | 2019-2021 | 2.8 (1.65 to 3.6) | 0.00 |
| **High fasting plasma glucose** | **Both** | |  |  | **Male** |  |  |  | **Female** |  |  |  |
|  | **Segment** | **Period** | **APC (95%CI)** | **p value** | **Segment** | **Period** | **APC (95%CI)** | **p value** | **Segment** | **Period** | **APC (95%CI)** | **p value** |
| **Deaths** | 1 | 1990-1995 | -0.07 (-0.43 to 0.27) | 0.60 | 1 | 1990-1995 | 0.02 (-0.23 to 0.26) | 0.895 | 1 | 1990-1995 | -0.07 (-0.37 to 0.22) | 0.54 |
|  | 2 | 1995-2000 | 3.58 (3.3 to 3.93) | 0.00 | 2 | 1995-2000 | 3.79 (3.57 to 4.02) | 0.00 | 2 | 1995-2000 | 3.58 (3.25 to 3.83) | 0.012 |
|  | 3 | 2000-2006 | -1.58 (-1.98 to -1.34) | 0.00 | 3 | 2000-2005 | -2.16 (-2.35 to -1.97) | 0.00 | 3 | 2000-2006 | -1.58 (-1.91 to -1.17) | 0.046 |
|  | 4 | 2006-2015 | 0.81 (0.65 to 1.03) | 0.00 | 4 | 2005-2015 | 1.16 (1.06 to 1.28) | 0.00 | 4 | 2006-2014 | 0.77 (-1.44 to 1.06) | 0.079 |
|  | 5 | 2015-2021 | -1.01 (-1.34 to -0.71) | 0.00 | 5 | 2015-2021 | -1.06 (-1.28 to -0.81) | 0.00 | 5 | 2014-2019 | -0.46 (-0.72 to 0.79) | 0.201 |
|  | 6 | … | … |  | 6 | … | … |  | 6 | 2019-2021 | -1.98 (-2.75 to -0.88) | 0.00 |
| **DALYs** | 1 | 1990-1995 | 0.49 (0.24 to 0.74) | 0.01 | 1 | 1990-1995 | 0.46 (0.18 to 0.71) | 0.030 | 1 | 1990-1995 | 0.58 (0.34 to 0.83) | 0.006 |
|  | 2 | 1995-2000 | 3.67 (3.46 to 3.9) | 0.00 | 2 | 1995-2000 | 3.85 (3.5 to 4.08) | 0.0008 | 2 | 1995-2000 | 3.66 (3.46 to 3.89) | 0.00 |
|  | 3 | 2000-2006 | -1.52 (-1.69 to -1.36) | 0.00 | 3 | 2000-2005 | -1.83 (-2.21 to -1.44) | 0.048 | 3 | 2000-2006 | -1.5 (-1.67 to -1.35) | 0.00 |
|  | 4 | 2006-2015 | 1.1 (0.97 to 1.23) | 0.00 | 4 | 2005-2008 | 0.79 (-1.47 to 1.41) | 0.222 | 4 | 2006-2015 | 1 (0.88 to 1.14) | 0.00 |
|  | 5 | 2015-2021 | -0.79 (-1.05 to -0.55) | 0.00 | 5 | 2008-2015 | 1.54 (-0.43 to 1.95) | 0.058 | 5 | 2015-2021 | -0.71 (-0.98 to -0.47) | 0.00 |
|  | 6 | … |  |  | 6 | 2015-2021 | -0.99 (-1.24 to -0.72) | 0.001 | 6 | … |  |  |
| **YLDs** | 1 | 1990-1996 | 2.47 (2.07 to 2.73) | 0.00 | 1 | 1990-1995 | 2.02 (1.63 to 2.31) | 0.00 | 1 | 1990-1996 | 2.65 (1.98 to 2.95) | 0.00 |
|  | 2 | 1996-2000 | 4.31 (3.91 to 4.93) | 0.00 | 2 | 1995-2000 | 4.28 (4.03 to 4.64) | 0.00 | 2 | 1996-2000 | 4.23 (3.74 to 4.9) | <0.001 |
|  | 3 | 2000-2005 | -1.63 (-2.06 to -1.35) | <0.001 | 3 | 2000-2005 | -1.68 (-1.94 to -1.45) | 0.00 | 3 | 2000-2005 | -1.59 (-2.23 to -1.26) | 0.002 |
|  | 4 | 2005-2010 | 0.71 (0.1 to 1.23) | 0.038 | 4 | 2005-2010 | 1.15 (0.73 to 1.47) | 0.001 | 4 | 2005-2010 | 0.6 (-0.2 to 1.24) | 0.088 |
|  | 5 | 2010-2015 | 2.33 (1.96 to 2.9) | 0.002 | 5 | 2010-2015 | 2.62 (2.36 to 3.02) | 0.00 | 5 | 2010-2015 | 2.29 (1.85 to 3.06) | 0.004 |
|  | 6 | 2015-2021 | -0.66 (-0.92 to -0.42) | 0.001 | 6 | 2015-2021 | -1 (-1.2 to -0.82) | 0.00 | 6 | 2015-2021 | -0.5 (-0.84 to -0.2) | 0.007 |
| **High body-mass index** | **Both** | |  |  | **Male** |  |  |  | **Female** |  |  |  |
|  | **Segment** | **Period** | **APC (95%CI)** | **p value** | **Segment** | **Period** | **APC (95%CI)** | **p value** | **Segment** | **Period** | **APC (95%CI)** | **p value** |
| **Deaths** | 1 | 1990-1993 | 17.96 (17.34 to 18.74) | 0.00 | 1 | 1990-1992 | 49.78 (47.59 to 52.11) | 0.00 | 1 | 1990-1993 | 14.96 (14.15 to 16.18) | 0.00 |
|  | 2 | 1993-1996 | 14.16 (13.2 to 15.16) | 0.00 | 2 | 1992-1995 | 27.34 (26.16 to 28.53) | 0.00 | 2 | 1993-1996 | 12.74 (9.92 to 13.39) | 0.00 |
|  | 3 | 1996-1999 | 11.27 (10.11 to 11.75) | 0.00 | 3 | 1995-1998 | 17.09 (15.69 to 18.01) | 0.00 | 3 | 1996-2003 | 9.77 (8.23 to 10.05) | 0.00 |
|  | 4 | 1999-2004 | 10.03 (8.21 to 10.3) | 0.00 | 4 | 1998-2003 | 12.98 (11.92 to 13.62) | 0.00 | 4 | 2003-2010 | 8.22 (7.36 to 8.57) | 0.00 |
|  | 5 | 2004-2010 | 8.38 (7.02 to 8.75) | 0.00 | 5 | 2003-2010 | 9.67 (9.12 to 10.21) | 0.00 | 5 | 2010-2021 | 7.14 (6.96 to 7.29) | 0.00 |
|  | 6 | 2010-2021 | 7.3 (7.03 to 7.58) | 0.00 | 6 | 2010-2021 | 7.84 (7.58 to 8.05) | 0.00 | 6 | … |  |  |
| **DALYs** | 1 | 1990-1993 | 17.96 (16.85 to 18.96) | 0.00 | 1 | 1990-1992 | 57.85 (54.79 to 61.05) | 0.00 | 1 | 1990-1993 | 14.58 (13.61 to 16.08) | 0.00 |
|  | 2 | 1993-1997 | 13.62 (12.82 to 14.33) | 0.00 | 2 | 1992-1995 | 28.73 (26.99 to 30.3) | 0.00 | 2 | 1993-1997 | 11.77 (10.29 to 12.49) | 0.00 |
|  | 3 | 1997-2004 | 10.38 (10.05 to 10.7) | 0.00 | 3 | 1995-1998 | 18.07 (15.48 to 19.23) | 0.000.00 | 3 | 1997-2004 | 9.55 (8.91 to 9.9) | 0.00 |
|  | 4 | 2004-2012 | 8.15 (7.89 to 8.49) | 0.00 | 4 | 1998-2003 | 13.49 (11.06 to 14.3) | 0.00 | 4 | 2004-2013 | 7.77 (7.49 to 8.06) | 0.00 |
|  | 5 | 2012-2021 | 7.05 (6.78 to 7.23) | 0.00 | 5 | 2003-2011 | 9.65 (8.93 to 10.27) | 0.00 | 5 | 2013-2021 | 6.73 (6.33 to 6.98) | 0.00 |
|  | 6 | … | … | … | 6 | 2011-2021 | 7.54 (7.11 to 7.88) | 0.00 | 6 | … |  |  |
| **YLDs** | 1 | 1990-1992 | 42.38 (39.88 to 44.97) | 0.00 | 1 | 1993-1995 | 430.08 (295.02 to 555.95) | 0.00 | 1 | 1990-1993 | 23.61 (22.39 to 24.87) | 0.00 |
|  | 2 | 1992-1995 | 24.73 (23.12 to 26.01) | 0.00 | 2 | 1995-2003 | 23.61 (17.12 to 40.82) | 0.00 | 2 | 1993-1997 | 15.63 (14.62 to 16.58) | 0.00 |
|  | 3 | 1995-1999 | 15.68 (14.53 to 16.94) | 0.00 | 3 | 2003-2021 | 9.65 (6.45 to 11.4) | 0.007 | 3 | 1997-2004 | 11.01 (10.54 to 11.55) | 0.00 |
|  | 4 | 1999-2004 | 11.91 (10.61 to 12.97) | 0.00 | 4 |  |  |  | 4 | 2004-2016 | 8.56 (8.38 to 8.8) | 0.00 |
|  | 5 | 2004-2014 | 9.18 (8.78 to 9.55) | 0.00 | 5 |  |  |  | 5 | 2016-2019 | 5.46 (4.68 to 6.9) | 0.00 |
|  | 6 | 2014-2021 | 7.35 (6.58 to 7.82) | 0.00 | 6 | … | … |  | 6 | 2019-2021 | 9.72 (7.65 to 11.18) | 0.00 |

**Abbreviations:** ASR, age-standardized rates; DALYs, disability-adjusted life-years; YLDs, years lived with disability; APC, annual percentage change; CI, confidence interval.
