# Supplementary material for: A GBD 2021 study of Alzheimer’s disease and other dementias attributable to metabolic risk factors and forecasts to 2045 in China
Source: Front Public Health. 2025 Apr 1;13:1575906. doi: 10.3389/fpubh.2025.1575906 (PMC11998917; doi:10.3389/fpubh.2025.1575906)
Supplement: Supplementary file 2 [file Supplementary_file_2.docx]

**Supplement 2 Table 1. Age effect** **of Alzheimer's disease and other dementias burden due to** **metabolic risk factors in China from 1990 to 2021**

| **Factor** | **Mortality in both sexes** | **Mortality in males** | **Mortality in females** |
| --- | --- | --- | --- |
|  | **RR (95% CI）** | **RR (95% CI）** | **RR (95% CI）** |
| **Age** |  |  |  |
| 40 to 44 | 1.02 ( 0.96 to 1.07 ) | 1.02 ( 0.94 to 1.1 ) | 1.01 ( 0.94 to 1.09 ) |
| 45 to 49 | 1.09 ( 1.04 to 1.14 ) | 1.09 ( 1.02 to 1.17 ) | 1.09 ( 1.02 to 1.16 ) |
| 50 to 54 | 1.17 ( 1.13 to 1.22 ) | 1.17 ( 1.1 to 1.24 ) | 1.17 ( 1.11 to 1.24 ) |
| 55 to 59 | 1.26 ( 1.22 to 1.3 ) | 1.25 ( 1.19 to 1.32 ) | 1.26 ( 1.21 to 1.32 ) |
| 60 to 64 | 1.35 ( 1.31 to 1.4 ) | 1.34 ( 1.28 to 1.41 ) | 1.36 ( 1.31 to 1.42 ) |
| 65 to 69 | 1.45 ( 1.41 to 1.5 ) | 1.44 ( 1.37 to 1.52 ) | 1.47 ( 1.41 to 1.53 ) |
| 70 to 74 | 1.56 ( 1.51 to 1.62 ) | 1.54 ( 1.46 to 1.64 ) | 1.58 ( 1.51 to 1.66 ) |
| 75 to 79 | 1.68 ( 1.61 to 1.75 ) | 1.66 ( 1.55 to 1.77 ) | 1.7 ( 1.61 to 1.8 ) |
| 80 to 84 | 1.8 ( 1.71 to 1.89 ) | 1.78 ( 1.64 to 1.92 ) | 1.84 ( 1.72 to 1.96 ) |
| 85 to 89 | 1.94 ( 1.83 to 2.05 ) | 1.9 ( 1.74 to 2.08 ) | 1.98 ( 1.83 to 2.14 ) |
| 90 to 94 | 2.08 ( 1.94 to 2.23 ) | 2.04 ( 1.84 to 2.26 ) | 2.13 ( 1.94 to 2.34 ) |
| 95 plus | 2.23 ( 2.07 to 2.41 ) | 2.19 ( 1.95 to 2.46 ) | 2.3 ( 2.07 to 2.55 ) |
| **Factor** | **DALYs in both sexes** | **DALYs in males** | **DALYs in females** |
|  | **RR (95% CI）** | **RR (95% CI）** | **RR (95% CI）** |
| **Age** |  |  |  |
| 40 to 44 | 1.01 ( 1 to 1.03 ) | 1.02 ( 1 to 1.03 ) | 1.01 ( 0.99 to 1.03 ) |
| 45 to 49 | 1.1 ( 1.08 to 1.12 ) | 1.1 ( 1.08 to 1.12 ) | 1.1 ( 1.08 to 1.12 ) |
| 50 to 54 | 1.2 ( 1.18 to 1.21 ) | 1.19 ( 1.17 to 1.21 ) | 1.2 ( 1.18 to 1.22 ) |
| 55 to 59 | 1.3 ( 1.28 to 1.32 ) | 1.29 ( 1.27 to 1.31 ) | 1.3 ( 1.28 to 1.33 ) |
| 60 to 64 | 1.41 ( 1.39 to 1.43 ) | 1.4 ( 1.38 to 1.42 ) | 1.42 ( 1.4 to 1.45 ) |
| 65 to 69 | 1.53 ( 1.51 to 1.56 ) | 1.51 ( 1.49 to 1.54 ) | 1.55 ( 1.52 to 1.58 ) |
| 70 to 74 | 1.67 ( 1.64 to 1.69 ) | 1.64 ( 1.61 to 1.67 ) | 1.69 ( 1.66 to 1.72 ) |
| 75 to 79 | 1.81 ( 1.77 to 1.84 ) | 1.78 ( 1.74 to 1.82 ) | 1.84 ( 1.8 to 1.88 ) |
| 80 to 84 | 1.96 ( 1.92 to 2.01 ) | 1.92 ( 1.87 to 1.97 ) | 2.01 ( 1.96 to 2.06 ) |
| 85 to 89 | 2.13 ( 2.08 to 2.18 ) | 2.08 ( 2.02 to 2.14 ) | 2.19 ( 2.13 to 2.25 ) |
| 90 to 94 | 2.32 ( 2.26 to 2.38 ) | 2.25 ( 2.19 to 2.33 ) | 2.38 ( 2.31 to 2.46 ) |
| 95 plus | 2.52 ( 2.45 to 2.59 ) | 2.44 ( 2.36 to 2.53 ) | 2.6 ( 2.51 to 2.68 ) |
| **Factor** | **YLDs in both sexes** | **YLDs in males** | **YLDs in females** |
|  | **RR (95% CI）** | **RR (95% CI）** | **RR (95% CI）** |
| **Age** |  |  |  |
| 40 to 44 | 1.02 ( 1 to 1.04 ) | 1.03 ( 1.01 to 1.05 ) | 1.01 ( 0.99 to 1.03 ) |
| 45 to 49 | 1.13 ( 1.11 to 1.15 ) | 1.13 ( 1.11 to 1.16 ) | 1.12 ( 1.1 to 1.14 ) |
| 50 to 54 | 1.25 ( 1.23 to 1.27 ) | 1.25 ( 1.23 to 1.27 ) | 1.25 ( 1.23 to 1.27 ) |
| 55 to 59 | 1.38 ( 1.36 to 1.41 ) | 1.38 ( 1.35 to 1.41 ) | 1.39 ( 1.36 to 1.41 ) |
| 60 to 64 | 1.53 ( 1.51 to 1.56 ) | 1.52 ( 1.49 to 1.55 ) | 1.54 ( 1.51 to 1.57 ) |
| 65 to 69 | 1.69 ( 1.66 to 1.73 ) | 1.67 ( 1.63 to 1.72 ) | 1.71 ( 1.68 to 1.74 ) |
| 70 to 74 | 1.87 ( 1.84 to 1.91 ) | 1.85 ( 1.8 to 1.9 ) | 1.9 ( 1.86 to 1.94 ) |
| 75 to 79 | 2.07 ( 2.03 to 2.12 ) | 2.04 ( 1.97 to 2.1 ) | 2.11 ( 2.06 to 2.16 ) |
| 80 to 84 | 2.29 ( 2.24 to 2.35 ) | 2.24 ( 2.17 to 2.32 ) | 2.34 ( 2.28 to 2.4 ) |
| 85 to 89 | 2.54 ( 2.47 to 2.61 ) | 2.47 ( 2.38 to 2.57 ) | 2.6 ( 2.52 to 2.67 ) |
| 90 to 94 | 2.81 ( 2.72 to 2.89 ) | 2.73 ( 2.61 to 2.85 ) | 2.88 ( 2.79 to 2.98 ) |
| 95 plus | 3.11 ( 3.01 to 3.21 ) | 3.01 ( 2.87 to 3.15 ) | 3.2 ( 3.09 to 3.31 ) |

RR donoted the rate ratio (RR) of Alzheimer's disease and other dementias mortality due to metabolic risk factors in different periods (period effects) and cohorts (cohort effects) relative to the reference points specified age and sex groups and calendar time periods.

CI, confidence interval

DALYs, Disability-Adjusted Life Years

YLDs, Years Lived with Disability

RR, rate ratio

**Supplement 2 Table 2. Period effect of Alzheimer's disease and other dementias burden due to metabolic risk factors in China from 1990 to 2021**

| **Factor** | **Mortality in both sexes** | **Mortality in males** | **Mortality in females** |
| --- | --- | --- | --- |
|  | **RR (95% CI）** | **RR (95% CI）** | **RR (95% CI）** |
| **Period** |  |  |  |
| 1992 to 1996 | 1 ( 1 to 1 ) | 1 ( 1 to 1 ) | 1 ( 1 to 1 ) |
| 1997 to 2001 | 1.18 ( 1.15 to 1.21 ) | 1.18 ( 1.14 to 1.23 ) | 1.19 ( 1.15 to 1.22 ) |
| 2002 to 2006 | 1.21 ( 1.18 to 1.25 ) | 1.2 ( 1.15 to 1.26 ) | 1.23 ( 1.18 to 1.27 ) |
| 2007 to 2011 | 1.29 ( 1.24 to 1.33 ) | 1.28 ( 1.21 to 1.35 ) | 1.3 ( 1.24 to 1.37 ) |
| 2012 to 2016 | 1.4 ( 1.34 to 1.47 ) | 1.4 ( 1.31 to 1.5 ) | 1.42 ( 1.33 to 1.51 ) |
| 2017 to 2021 | 1.47 ( 1.4 to 1.55 ) | 1.45 ( 1.34 to 1.57 ) | 1.5 ( 1.39 to 1.61 ) |
| **Factor** | **DALYs in both sexes** | **DALYs in males** | **DALYs in females** |
|  | **RR (95% CI）** | **RR (95% CI）** | **RR (95% CI）** |
| **Period** |  |  |  |
| 1992 to 1996 | 1 ( 1 to 1 ) | 1 ( 1 to 1 ) | 1 ( 1 to 1 ) |
| 1997 to 2001 | 1.2 ( 1.18 to 1.21 ) | 1.19 ( 1.18 to 1.21 ) | 1.2 ( 1.19 to 1.22 ) |
| 2002 to 2006 | 1.25 ( 1.23 to 1.26 ) | 1.24 ( 1.22 to 1.25 ) | 1.26 ( 1.24 to 1.28 ) |
| 2007 to 2011 | 1.33 ( 1.31 to 1.35 ) | 1.32 ( 1.3 to 1.34 ) | 1.34 ( 1.32 to 1.36 ) |
| 2012 to 2016 | 1.48 ( 1.45 to 1.5 ) | 1.47 ( 1.44 to 1.49 ) | 1.49 ( 1.47 to 1.52 ) |
| 2017 to 2021 | 1.55 ( 1.53 to 1.58 ) | 1.52 ( 1.49 to 1.55 ) | 1.58 ( 1.55 to 1.61 ) |
| **Factor** | **YLDs in both sexes** | **YLDs in males** | **YLDs in females** |
|  | **RR (95% CI）** | **RR (95% CI）** | **RR (95% CI）** |
| **Period** |  |  |  |
| 1992 to 1996 | 1 ( 1 to 1 ) | 1 ( 1 to 1 ) | 1 ( 1 to 1 ) |
| 1997 to 2001 | 1.23 ( 1.21 to 1.25 ) | 1.23 ( 1.21 to 1.25 ) | 1.24 ( 1.22 to 1.26 ) |
| 2002 to 2006 | 1.3 ( 1.28 to 1.32 ) | 1.29 ( 1.26 to 1.31 ) | 1.31 ( 1.29 to 1.33 ) |
| 2007 to 2011 | 1.39 ( 1.37 to 1.41 ) | 1.39 ( 1.36 to 1.42 ) | 1.4 ( 1.38 to 1.42 ) |
| 2012 to 2016 | 1.6 ( 1.58 to 1.63 ) | 1.6 ( 1.56 to 1.63 ) | 1.62 ( 1.59 to 1.65 ) |
| 2017 to 2021 | 1.71 ( 1.68 to 1.74 ) | 1.66 ( 1.62 to 1.71 ) | 1.74 ( 1.71 to 1.78 ) |

RR donoted the rate ratio (RR) of Alzheimer's disease and other dementias mortality due to metabolic risk factors in different periods (period effects) and cohorts (cohort effects) relative to the reference points specified age and sex groups and calendar time periods.

DALYs, Disability-Adjusted Life Years

YLDs, Years Lived with Disability

RR, rate ratio

CI, confidence interval

**Supplement 2 Table 3. Cohort effect of Alzheimer's disease and other dementias burden due to metabolic risk factors in China from 1990 to 2021**

| **Factor** | **Mortality in both sexes** | **Mortality in males** | **Mortality in females** |
| --- | --- | --- | --- |
|  | **RR (95% CI）** | **RR (95% CI）** | **RR (95% CI）** |
| **Cohort** |  |  |  |
| 1892 to 1901 | 0.52 ( 0.45 to 0.59 ) | 0.51 ( 0.36 to 0.73 ) | 0.51 ( 0.44 to 0.59 ) |
| 1897 to 1906 | 0.55 ( 0.52 to 0.58 ) | 0.55 ( 0.48 to 0.62 ) | 0.54 ( 0.51 to 0.58 ) |
| 1902 to 1911 | 0.58 ( 0.56 to 0.61 ) | 0.58 ( 0.54 to 0.63 ) | 0.57 ( 0.54 to 0.6 ) |
| 1907 to 1916 | 0.61 ( 0.59 to 0.63 ) | 0.62 ( 0.58 to 0.65 ) | 0.6 ( 0.57 to 0.63 ) |
| 1912 to 1921 | 0.64 ( 0.61 to 0.66 ) | 0.65 ( 0.61 to 0.68 ) | 0.62 ( 0.6 to 0.65 ) |
| 1917 to 1926 | 0.67 ( 0.64 to 0.69 ) | 0.68 ( 0.64 to 0.72 ) | 0.66 ( 0.63 to 0.69 ) |
| 1922 to 1931 | 0.7 ( 0.68 to 0.73 ) | 0.71 ( 0.68 to 0.75 ) | 0.7 ( 0.67 to 0.73 ) |
| 1927 to 1936 | 0.74 ( 0.71 to 0.76 ) | 0.75 ( 0.71 to 0.79 ) | 0.73 ( 0.7 to 0.76 ) |
| 1932 to 1941 | 0.78 ( 0.75 to 0.8 ) | 0.79 ( 0.75 to 0.83 ) | 0.78 ( 0.74 to 0.81 ) |
| 1937 to 1946 | 0.84 ( 0.81 to 0.86 ) | 0.84 ( 0.8 to 0.88 ) | 0.84 ( 0.81 to 0.88 ) |
| 1942 to 1951 | 0.92 ( 0.89 to 0.95 ) | 0.91 ( 0.87 to 0.96 ) | 0.92 ( 0.88 to 0.96 ) |
| 1947 to 1956 | 1 ( 1 to 1 ) | 1 ( 1 to 1 ) | 1 ( 1 to 1 ) |
| 1952 to 1961 | 1.1 ( 1.05 to 1.15 ) | 1.11 ( 1.03 to 1.19 ) | 1.1 ( 1.03 to 1.17 ) |
| 1957 to 1966 | 1.23 ( 1.15 to 1.32 ) | 1.22 ( 1.1 to 1.36 ) | 1.24 ( 1.13 to 1.37 ) |
| 1962 to 1971 | 1.39 ( 1.23 to 1.57 ) | 1.37 ( 1.14 to 1.63 ) | 1.42 ( 1.2 to 1.68 ) |
| 1967 to 1976 | 1.59 ( 1.19 to 2.11 ) | 1.52 ( 1.01 to 2.29 ) | 1.59 ( 1.06 to 2.39 ) |
| 1972 to 1981 | 1.72 ( 0.57 to 5.16 ) | 1.68 ( 0.36 to 7.98 ) | 1.95 ( 0.4 to 9.46 ) |
| **Factor** | **DALYs in both sexes** | **DALYs in males** | **DALYs in females** |
|  | **RR (95% CI）** | **RR (95% CI）** | **RR (95% CI）** |
| **Cohort** |  |  |  |
| 1892 to 1901 | 0.47 ( 0.42 to 0.53 ) | 0.48 ( 0.39 to 0.59 ) | 0.47 ( 0.42 to 0.52 ) |
| 1897 to 1906 | 0.5 ( 0.48 to 0.53 ) | 0.51 ( 0.47 to 0.54 ) | 0.5 ( 0.47 to 0.52 ) |
| 1902 to 1911 | 0.53 ( 0.52 to 0.55 ) | 0.54 ( 0.52 to 0.56 ) | 0.53 ( 0.51 to 0.54 ) |
| 1907 to 1916 | 0.57 ( 0.55 to 0.58 ) | 0.57 ( 0.56 to 0.59 ) | 0.55 ( 0.54 to 0.56 ) |
| 1912 to 1921 | 0.59 ( 0.58 to 0.6 ) | 0.6 ( 0.59 to 0.62 ) | 0.58 ( 0.57 to 0.59 ) |
| 1917 to 1926 | 0.62 ( 0.61 to 0.63 ) | 0.64 ( 0.63 to 0.65 ) | 0.61 ( 0.6 to 0.62 ) |
| 1922 to 1931 | 0.66 ( 0.65 to 0.67 ) | 0.68 ( 0.66 to 0.69 ) | 0.65 ( 0.64 to 0.66 ) |
| 1927 to 1936 | 0.7 ( 0.69 to 0.71 ) | 0.72 ( 0.71 to 0.73 ) | 0.7 ( 0.68 to 0.71 ) |
| 1932 to 1941 | 0.75 ( 0.74 to 0.76 ) | 0.76 ( 0.75 to 0.77 ) | 0.75 ( 0.74 to 0.76 ) |
| 1937 to 1946 | 0.82 ( 0.81 to 0.83 ) | 0.82 ( 0.8 to 0.83 ) | 0.82 ( 0.81 to 0.84 ) |
| 1942 to 1951 | 0.9 ( 0.89 to 0.92 ) | 0.9 ( 0.89 to 0.91 ) | 0.91 ( 0.9 to 0.92 ) |
| 1947 to 1956 | 1 ( 1 to 1 ) | 1 ( 1 to 1 ) | 1 ( 1 to 1 ) |
| 1952 to 1961 | 1.11 ( 1.09 to 1.13 ) | 1.12 ( 1.09 to 1.14 ) | 1.11 ( 1.09 to 1.13 ) |
| 1957 to 1966 | 1.27 ( 1.23 to 1.3 ) | 1.26 ( 1.22 to 1.29 ) | 1.28 ( 1.24 to 1.32 ) |
| 1962 to 1971 | 1.45 ( 1.4 to 1.51 ) | 1.42 ( 1.36 to 1.47 ) | 1.49 ( 1.42 to 1.56 ) |
| 1967 to 1976 | 1.67 ( 1.55 to 1.81 ) | 1.62 ( 1.5 to 1.76 ) | 1.73 ( 1.57 to 1.9 ) |
| 1972 to 1981 | 2.03 ( 1.59 to 2.6 ) | 1.93 ( 1.51 to 2.47 ) | 2.15 ( 1.58 to 2.92 ) |
| **Factor** | **YLDs in both sexes** | **YLDs in males** | **YLDs in females** |
|  | **RR (95% CI）** | **RR (95% CI）** | **RR (95% CI）** |
| **Cohort** |  |  |  |
| 1892 to 1901 | 0.38 ( 0.31 to 0.45 ) | 0.4 ( 0.27 to 0.58 ) | 0.37 ( 0.31 to 0.43 ) |
| 1897 to 1906 | 0.41 ( 0.39 to 0.44 ) | 0.42 ( 0.38 to 0.47 ) | 0.41 ( 0.38 to 0.43 ) |
| 1902 to 1911 | 0.45 ( 0.43 to 0.46 ) | 0.45 ( 0.43 to 0.48 ) | 0.44 ( 0.43 to 0.45 ) |
| 1907 to 1916 | 0.48 ( 0.47 to 0.5 ) | 0.49 ( 0.48 to 0.51 ) | 0.47 ( 0.46 to 0.49 ) |
| 1912 to 1921 | 0.52 ( 0.51 to 0.53 ) | 0.53 ( 0.51 to 0.54 ) | 0.51 ( 0.5 to 0.52 ) |
| 1917 to 1926 | 0.55 ( 0.54 to 0.57 ) | 0.57 ( 0.55 to 0.58 ) | 0.55 ( 0.53 to 0.56 ) |
| 1922 to 1931 | 0.6 ( 0.59 to 0.61 ) | 0.61 ( 0.6 to 0.63 ) | 0.59 ( 0.58 to 0.6 ) |
| 1927 to 1936 | 0.65 ( 0.64 to 0.66 ) | 0.67 ( 0.65 to 0.68 ) | 0.65 ( 0.64 to 0.66 ) |
| 1932 to 1941 | 0.71 ( 0.7 to 0.72 ) | 0.72 ( 0.71 to 0.74 ) | 0.71 ( 0.7 to 0.73 ) |
| 1937 to 1946 | 0.79 ( 0.78 to 0.8 ) | 0.79 ( 0.77 to 0.8 ) | 0.8 ( 0.78 to 0.81 ) |
| 1942 to 1951 | 0.88 ( 0.87 to 0.9 ) | 0.88 ( 0.86 to 0.9 ) | 0.89 ( 0.88 to 0.91 ) |
| 1947 to 1956 | 1 ( 1 to 1 ) | 1 ( 1 to 1 ) | 1 ( 1 to 1 ) |
| 1952 to 1961 | 1.13 ( 1.11 to 1.15 ) | 1.14 ( 1.11 to 1.17 ) | 1.13 ( 1.1 to 1.15 ) |
| 1957 to 1966 | 1.31 ( 1.27 to 1.34 ) | 1.29 ( 1.25 to 1.34 ) | 1.31 ( 1.27 to 1.35 ) |
| 1962 to 1971 | 1.52 ( 1.46 to 1.58 ) | 1.47 ( 1.41 to 1.54 ) | 1.55 ( 1.49 to 1.62 ) |
| 1967 to 1976 | 1.76 ( 1.64 to 1.89 ) | 1.7 ( 1.57 to 1.84 ) | 1.83 ( 1.68 to 1.98 ) |
| 1972 to 1981 | 2.13 ( 1.73 to 2.63 ) | 2.01 ( 1.59 to 2.54 ) | 2.28 ( 1.79 to 2.9 ) |

RR donated the rate ratio (RR) of Alzheimer's disease and other dementias DALYs rate due to metabolic risk factors in different periods (period effects) and cohorts (cohort effects) relative to the reference points specified age and sex groups and calendar time periods.

CI, confidence interval

DALYs, Disability-Adjusted Life Years

YLDs, Years Lived with Disability

RR, rate ratio
